# Evaluating the contributions of top-down and bottom-up processing on eye movements during parallel visual search

**DOI:** 10.3758/s13414-025-03199-z

**Published:** 2026-02-23

**Authors:** Howard Jia He Tan, Alejandro Lleras, Zoe Jing Xu, Yifan Ding, Simona Buetti

**Affiliations:** University of Illinois at Urbana-Champaign, 603 E. Daniel St., Champaign, IL 61820 USA

## Abstract

In the current study, we used an efficient visual search paradigm in a pseudo-realistic environment, with well-controlled search stimuli that allow a simultaneous evaluation of the impact of top-down and bottom-up factors on eye-movement patterns. Our stimuli varied along the color dimension to manipulate target-distractor similarity and our displays contained a salient stimulus of higher salience than target and other less-salient distractor stimuli. We manipulated task instructions, introducing a free-view instruction condition to serve as a baseline for how bottom-up contrast guided eye movements in one group of participants, and a top-down search instruction in a second group, where subjects were asked to find the red target in the scene. Experiment 1 assessed the impact of set size of less-salient distractors across both instructions. Experiment 2 examined target-distractor similarity effects for the less-salient distractors. We compared the likelihood that the first fixation in a trial would be selective towards the target (top-down) versus the high-salience singleton (bottom-up) and studied how this selectivity varied as a function of initial saccade latency. Interestingly, the results from the free-view conditions showed selectivity for the high-salience item during the first fixation was sustained across saccade latencies, yet the high-salience items capture very few saccades in the search task, suggesting attention might be in limbo early in the trial. Indeed, the results also showed that it takes time for saccades to be correctly directed at the target in a search task.

## Introduction

We make eye movements daily and they provide us with valuable insights into attentional and visual perceptual processes. Our eyes saccade and fixate to gather information from our surroundings and this visual information is used in processes like object recognition, scene perception, and visual search. Often, we interpret these saccades as overt shifts of attention, aiding us in collecting high spatial frequency visual information through the fovea (e.g., McCarley & Kramer, 2007; Posner, 1980). From our everyday experiences, it becomes evident that we move our gaze in different ways, driven by the diverse range of tasks we engage in. These task demands can influence the spatial and temporal parameters of our eye movements, affecting factors such as saccade amplitude and fixation duration (e.g., Mills et al., 2011).

One common visual task in our daily lives is visual search, and models describing search behavior frequently invoke the concepts of top-down and bottom-up processing. Information is gathered from the visual properties of the stimulus in a bottom-up manner (e.g., Itti & Koch, 2000; Koch & Ullman, 1985; Treisman & Gelade, 1980). Unique stimuli characteristics within a feature dimension like color or shape can attract attention (Treisman & Gelade, 1980). These stimuli characteristics can also affect the guidance of eye movements in a bottom-up manner (Koch & Ullman, 1985; Theeuwes et al., 1999). Itti and Koch (2000) proposed that saliency in the form of a local contrast in features can predict movements of attention. And multiple studies have shown evidence for bottom-up attentional capture by salient singletons in the absence of search strategies (e.g., Jonides & Yantis, 1988; Theeuwes, 1992; Theeuwes, 2004).

Attentional deployment in a scene can also be influenced by top-down knowledge. In a visual search task for example, objects in the scene are compared to a target template (e.g., Buetti et al., 2016; Duncan & Humphreys, 1989; Wolfe, 2021; Zelinsky, 2008). There is now a consensus view that bottom-up saliency is not the sole driver of eye movements and eye fixations are not solely predicted by image statistics (e.g., Awh et al., 2012; Henderson et al., 2009; Henderson et al., 2007). For example, knowing the target of a search task through a top-down experimental manipulation leads to more saccades towards the target and away from salient singleton distractors (Chen & Zelinsky, 2006).

Researchers have developed computational models to quantify these effects of the bottom-up stimulus properties and top-down knowledge in guiding eye movements. Itti and Koch (2000) first investigated the use of bottom-up saliency maps that computed localized center-surround differences in images as a predictor of eye fixations. Torralba et al. (2006) modeled eye fixations in search task conditions by combining local bottom-up saliency with scene contexts derived from global image features and spatial associations. Navalpakkam and Itti (2005) incorporated the addition of top-down guidance maps based on a visual memory of the target. A prominent model of eye movements during search, the Target Acquisition Model (Zelinsky, 2008), simulates eye-movement behavior in target-present scenes using top-down target maps computed by comparing the visual similarity between the target representation along 27 visual features and the search scene. Notably, these image-based, computational models used complex, real-world scenes as stimuli, and the metrics of saliency computed for these stimuli varied across models. However, efforts have been made to establish a standardized benchmark for these computational models (Bylinskii et al., 2015; Bylinskii et al., 2018). In contrast, studies focused on determining the impact of salient objects in a scene on attentional deployment have, by and large, used simplified and well-controlled displays (e.g., Folk & Remington, 1998; Gaspelin et al., 2017; Theeuwes, 1992; van Zoest et al., 2004; Van Zoest & Donk, 2008; but see Kotseruba et al., 2020).

The goal of the present study was to determine the contribution of top-down attentional modulations and bottom-up local contrast computations in driving eye-movement patterns during visual search in the presence of a salient element in a scene, using stimuli that are more naturalistic and complex than those that have been used in this literature in the past.

van Zoest et al. (2004) investigated the contribution of top-down and bottom-up in eye movements using oriented line-segment displays (Experiments 1 and 2) and a color distractor among oriented lines (Experiment 3) to explore the effect of bottom-up attentional capture in the presence of top-down signals. Their subjects had to search for singleton targets amongst less-salient homogeneous distractors, and some trials would also have a more-salient singleton distractor. van Zoest et al. (2004) found that the fastest saccades were more driven by bottom-up information, being more often directed towards the singleton salient distractor, whereas slower saccades tended to be driven by top-down information, being more often directed towards the target. However, the authors noted that the distinction between bottom-up and top-down control of saccades was not clear: saccades that appeared to be driven by bottom-up information could still be driven by a top-down search goal if subjects were confusing the target for the singleton distractor. This was a possible concern in van Zoest et al. (2004) since the target was defined as a slanted line (Experiment 1), and the singleton distractor was a mirrored slanted line, where the two may be sharing the feature space being used for the search task. van Zoest et al. (2004) control for this target confusability issue in their Experiment 2 explicitly, and further avoid this issue by using the color dimension to define target and distractor (Experiment 3). Van Zoest and Donk (2008) later showed that when targets and distractors are both defined by color instead, goal-driven selection can occur very early in processing. Participants in their study could efficiently select a red target even in the presence of a salient green distractor, suggesting that color information allows for earlier top-down control.

**Fig. 1:**
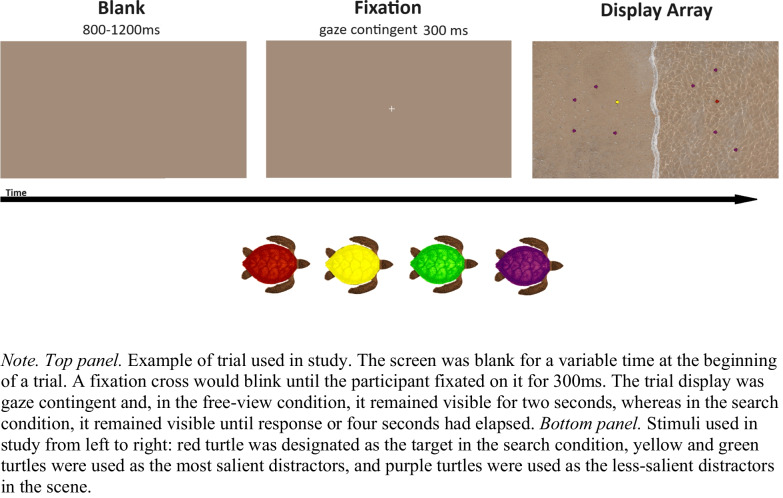
Example of display stimuli used in the study

In the present study, we address the target confusability issue by using the color dimension as in Van Zoest and Donk (2008), reducing confusability by using colors that share no overlap between the target and salient singleton distractor (see Schurgin et al., 2020). Additionally, the displays used by van Zoest and colleagues consisted of densely packed line segments arranged in a grid pattern, where the “nontarget” elements effectively formed a textured background. This design made it difficult to isolate the contribution of bottom-up factors like salience to eye movements, as saccades were only classified as landing on either the target or the salient distractor, with all other saccades discarded from analysis. To more clearly quantify the effects of both top-down and bottom-up influences on eye movements, we constructed displays with well-separated stimuli that minimized crowding and textural effects (Fig. 1).

Furthermore, these previous studies did not include a condition aimed at measuring the base-rate attentional attraction of the salient distractor, in other words, the bottom-up pull of this item in their display, absent a systematic top-down goal.

To address this, we implemented a between-subjects instruction manipulation to compare eye-movement performance under a systematic top-down goal (the search condition) to performance in the absence of a systematic top-down goal (the free-view condition). In the search condition, participants were instructed to search for the red turtle. In the free-view condition, participants were told to freely view the scene, with no further instructions than that. This condition allowed us to assess the base-rate, or baseline, for how bottom-up factors affect eye movements, in the absence of any systematic top-down goals. We expected that the two task demands would incur different eye-movement patterns (e.g., Mills et al., 2011; Yarbus, 1967).

In the free-view condition, subjects might simply look at the object that “pops out” the most in the visual display, in this case the salient turtle. In that sense, because there is an absence of top-down task modulation in the free-view condition (or at least the absence of a consistent top-down goal across participants), systematic eye-movement patterns can then be primarily attributed to bottom-up factors. In contrast, during search, the explicit goal of finding the red turtle allows us to examine how these same display manipulations interact with top-down control.

We attempted to quantify the effects of top-down and bottom-up factors in driving eye movements by examining two well-studied properties of visual search: distractor set size and target-distractor similarity (e.g., Buetti et al., 2016; Lleras et al., 2020). In the top-down search condition, the target is known to be the red turtle, and we expected observers to take longer to reject items as non-targets as target-distractor similarity increases (e.g., Buetti et al., 2016; Duncan & Humphreys, 1989; Lleras et al., 2020). Similarly, the time to find the target ought to also depend on the number of distractors in the display. We should be able to observe these trends in the search condition. Additionally, because identical displays were viewed under both instruction conditions, differences in how set size or color similarity affect eye movements between conditions can then be attributed to the presence or absence of top-down control, rather than to differences in bottom-up stimulus properties.

It is important to note that our approach differs from traditional attentional capture studies that measure rapid bottom-up selection. Rather than examining automatic capture by salient items during goal-directed search, we aimed to understand how stimulus properties influence viewing patterns both with and without explicit search goals. While free-viewing behavior can be influenced by multiple factors including scene semantics and viewing biases (e.g., Henderson & Hayes, 2017; Kiat et al., 2022; Peacock et al., 2020; Tatler et al., 2011), it remains a valuable baseline condition as it allows us to observe viewing patterns without explicit task goals. This approach is widely used in computational modeling and eye-movement research to examine how stimulus properties influence viewing behavior (Borji & Itti, 2015; Harel et al., 2007; Jiang et al., 2015; Judd et al., 2012). This approach remains the standard way of eliciting eye-movement data to create the databases used to test saliency models.

By comparing how these manipulations affect viewing patterns differently between free-viewing and search conditions, we can better understand how bottom-up stimulus properties interact with top-down goals. While free viewing cannot completely isolate bottom-up influences from other factors that guide attention, it still provides a useful baseline for comparing against goal-directed search behavior (Castelhano et al., 2009; Dodd et al., 2009; Mills et al., 2011).

Following the design of Van Zoest and Donk (2008), each of our displays included a red turtle (the target in the search condition), a more-salient singleton turtle stimulus, which could be either yellow or green, as well as less-salient turtles (purple in Experiment 1; purple or blue in Experiment 2). We included a salient distractor in every display, while randomly changing its color, so that subjects would be unable to anticipate its color on any trial. This color-varying manipulation is known to minimize any potential contributions from distractor suppression mechanisms, which work to minimize the attentional pull from a salient, but constant stimulus (e.g., Gaspelin et al., 2017). Importantly, the salient turtles we used had higher luminance and should be more salient than the red turtle, with the goal that it would consistently compete for attentional selection in a bottom-up fashion, hopefully being the most frequently fixated item in the display in the free-view condition.

We studied different hypotheses with regard to how bottom-up factors impact eye movement in our more naturalistic scenes. If bottom-up driven fixations are only impacted by detection of the most salient items in a display (red, yellow, or green) in a winner-take-all fashion as described in Koch and Ullman (1985) and by later derivative models (e.g., Itti & Koch, 2000), then the properties of less-salient items should not impact first fixation selectivity for the item with the highest attentional priority in either condition. Note that the winner-take-all mechanism does not require that the most salient item should attract all first fixations because even at the top of the salience scale there is likely to be encoding noise. That said, the selection should take place among the most salient items in the display, and locations containing significantly less salient items would not affect that selection. We refer to this prediction as the *Winner-take-all Hypothesis* in this study. Alternatively, according to the Normalization Theory of Attention, the bottom-up pull of any one item in a scene is relative to other information in the display, with each item in the display having a probability of capturing attention that is relative to the sum of all items’ salience (Reynolds & Heeger, 2009). Assuming there are *N* items *i* in the display, each with salience *S*(*i*), the probability of any item being visually selected first in a trial may be thought of in the following form:

1 $$p(i)= \frac{{S}_{i}}{{\sum }_{i}^{N}{S}_{i}}$$

where ${\sum }_{i}^{N}p(i)= 1$. Following Equation 1, an item is not necessarily ignored because it is less salient, it is just less likely to capture attention than a more salient item. Factors like the number of items in the scene, as well as the bottom-up contrast of each item in the display, should all end up impacting the probability of moving the eyes to the item with the highest salience value. As a result, we would expect that first fixation selectivity for the salient turtle will decrease as a function of the total set size since the underlying image statistics are being changed as we introduce more purple turtles into the display. As a result, the probability that the first fixation goes towards any one of the purple turtles ought to increase as the number of purple turtles increases, in spite of the fact that the purple turtles are the least salient items in the scene. We refer to this prediction as the *Probabilistic Salience Hypothesis* in this study.

With regard to the top-down theories of visual search, we aimed to characterize how typical visual search manipulations affect fixation selectivity, when in the presence of a demonstrably high-salience item. In terms of color similarity, to define the salient turtle, we selected very dissimilar colors (yellow or green) to the red used to define the target turtle. Furthermore, to define the less salient turtles, we selected a color (purple) that was somewhat more similar (sharing a red hue) to the color of the target to create a situation where increasing numbers of purple turtles would lead to longer times to find the red turtle (e.g., Buetti et al., 2016; Lleras et al., 2020). Importantly, the red-purple difference was sufficiently large so that this difference could be detected in parallel across the display (for a review, see Lleras, Buetti & Xu, 2022). As a basic validation, we would expect that when performing the search task for the red turtle, increasing set size or target-distractor similarity for these less-salient purple turtles should lower first fixation selectivity for the target, reflecting the increase in search difficulty (e.g., Buetti et al., 2016; Lleras et al., 2020). More critically, if target-distractor similarity is indeed the primary driver of attentional selection as predicted by template guidance theories, then we should observe more first fixations towards the less-salient purple distractors than the salient yellow or green distractor, since purple shares more features with red than yellow or green does. We refer to this set of predictions as the *Template Guidance Hypothesis* in this study.

Finally, we also examined the temporal dynamics for how top-down and bottom-up factors impact fixation selectivity in visual search, inspired by the analyses pioneered by van Zoest et al. (2004). The timing account of visual selection supposes that both stimulus-driven and goal-driven control contribute to eye movements independently and operate in different time windows. Thus, the selectivity of the first fixation in search would evolve as a function of time: the fastest saccades ought to be driven by bottom-up salience and thus be more likely to land on the salient turtle, whereas slower saccades ought to be more driven by top-down factors and be more likely to land on the search target. Along those lines, van Heusden et al. (2022) proposed the concept of attentional limbo, a time period whereby saccadic selectivity is neither affected by an item’s salience nor by its goal relevance. This would manifest as a period of non-selectivity for saccades, and would occur during the shift from fast, bottom-up driven saccades to slower, goal-driven saccades. We refer to this set of predictions as the *Temporal Dynamic Hypothesis*.

## Experiment 1

In Experiment 1, we presented visual displays with one red turtle, one yellow or green salient turtle, and a varying number of less-salient purple turtles. In the search condition, subjects were asked to search for the red turtle. In the free-view condition, subjects were asked to view the scene freely.

### Method

The methods and experimental protocols were approved by the Institutional Review Board at the University of Illinois, Urbana-Champaign (UIUC), and are in accordance with the Declaration of Helsinki. In all the experiments, participants were prescreened for normal or corrected-to normal visual acuity in a subject pool questionnaire and screened for normal color vision with Ishihara color plates. All relevant data and codes have been made accessible via the Open Science Framework (link: https://osf.io/rjsbu/).

#### Participants

Forty-six participants were recruited from the subject pool for Experiment 1 at the UIUC in exchange for course credit or $8. Informed consent was obtained from all participants. Due to experimenter error, demographic information was not correctly collected. The recruiting process ensured that participants ranged from 18–52 years old, most of them undergraduate students from UIUC. Participants were excluded if data were only obtained on less than half of trials, usually as a result of poor eye tracking. One participant in the free-view condition was excluded because they did not meet the data criterion. Participants in the search condition were excluded if their accuracy was below 85% (group mean accuracy =.97 and SDs = 0.03), or if they had mean response times more than 2.5 standard deviations away from the group average mean response time (group average mean response time = 766.66 ms, SD = 63.58 ms). One participant in the search condition was excluded because they did not meet the accuracy criterion. Four participants’ data contained no usable eye-tracking information due to experimenter error. Forty participants’ data were included in the analysis in Experiment 1. Based on a pilot study (N = 32), we determined that 24 participants were sufficient to detect the differences between the two instruction conditions in terms of first fixations to the red turtle (Cohen’s *d* = 1.56) and impact of low salience items on first fixations to the salient turtle in the free-viewing condition (Cohen’s *f* =.721). With α =.05 (two-tailed), a sample of 24 participants would provide 95% power to detect an effect size of *d* = 1.56 and greater than 99% power for *f* =.721. However, the final sample size of 40 participants was chosen to match Experiment 2.

#### Apparatus

The experiment was programmed in Python with Psychopy 2.0 and ran on 64-bit Windows10 PCs. Eye-tracker related control functions were imported from SR-Research documentation. Stimuli were presented on a gamma-corrected 24-in. LCD monitor set at a 239.75-Hz refresh rate with a 1,920 × 1,080 resolution. Eye movements were recorded with a SR-Research Eyelink 1000 Plus desktop mount eye tracker sampling at 1,000 Hz. Participants were seated 91 cm away from the monitor and so the display occupieds approximately 32° and 19° of visual angle. Participants used a chin and forehead rest to minimize head movements. Eye-movement data were collected for either eye, or from both eyes depending on how noisy a participant’s data was, and only one eye’s data were used in the analysis. Saccades were defined using the Eyelink 1000 Plus’s default velocity and acceleration thresholds (30°/s and 8,000°/s^2^). Fixations were defined by Eyelink 1000 Plus’s default inter-saccade periods.

#### Stimuli

The stimuli were colored turtles facing either left or right, presented on a beach background (Fig. 1). The stimuli were 35 × 30 pixels (.61° ×.52° in visual angle). In order of lightness, the yellow salient turtle was defined at HSL (hue, saturation, lightness) values of (59, 100%, 33%), the green salient turtle at (113, 99%, 28%), the red turtle at (4, 100%, 22%), and purple less-salient turtles at (307, 77%, 17%). The corresponding average RGB values were: yellow (168, 165, 0), green (17, 142, 1), red (112, 7, 0), purple (77, 10, 69). The yellow and green colors chosen had the highest luminance by design, and both were employed as the salient color (across individual trials) to minimize distractor suppression. Although our stimuli were hand selected, we have validated their intended level of salience through the use of the GBVS saliency toolbox (Harel et al., 2007). This analysis can be found in Appendix A.

Stimuli were placed onto the display in a rectangular grid with jitter added. Stimuli positions for a particular trial were randomly generated once and all participants saw the same arrangement of stimuli for that particular trial. The average eccentricity of the turtle stimuli from the center was 8.68° in visual angle, with a range of up to 11.23° horizontally and 5.44° vertically (total range of 10.89°). The stimuli were also placed with a minimum distance from the center fixation cross to be able to capture meaningful eye movements. The red turtle was placed on either the left or right side of the display and the salient turtle was always placed on the opposite side. The red turtle and salient turtle were always placed on opposite sides of the display to avoid center-of-gravity saccades that could occur if they were placed closer together. This separation ensured that first saccades would more clearly indicate which stimulus was being targeted, providing more discriminating data regarding attentional selection. The beach background contained a wave in the center, and contained a water light reflection pattern on either the left or the right side. Every background in the experiment that was not a trial display, including calibration, had the same average color as the beach background to minimize pupil size changes.

#### Design

There was a between-subjects manipulation of instruction (free-view, search) and subjects were randomly assigned to an instruction condition. Both instruction conditions received the same within-subject manipulations and the exact same generated search arrays. There were five set sizes for the less-salient purple turtles (0, 2, 4, 8, 16) and two salient turtle colors (yellow, green). Red turtle orientation (facing left, right) and background orientation (water reflection pattern on left, right) were also controlled for, resulting in 40 conditions (2 × 2 × 2 × 5) that were repeated six times, summing up to a total of 240 trials. The trials were presented in a blocked design, where all 40 conditions were presented once, randomly, in a block and in random order across subjects. For analysis, the red turtle orientation and background orientation were collapsed. Preliminary analyses showed background orientation did not systematically affect first fixation patterns during free viewing. The center wave itself drew relatively few first fixations (<10% on average) across both conditions.

#### Procedure

The eye tracker was calibrated using a 3 × 3 spatial array before the experiment, and a single-point calibration (drift correct) occurred between every block of trials. Subjects were required to have a maximum calibration error of less than 1° for the center point and less than 1.5° in any of the other eight points.

Subjects in the free-view condition were given the following instructions: “In this experiment, we are interested in studying how people explore images with their eyes. We will show you several images and we just want you to look around. We will record your eye movements so that we can study how you are exploring the images.”

Subjects in the search condition were given the following instructions: “This experiment requires you to visually search for a target object and make a decision about it. The target you will be searching for is a red turtle. Your task is to decide towards which side the turtle’s head is facing, by pressing the LEFT or RIGHT ARROW key on the button box. And please try to respond as fast and as accurately as possible.” Subjects in the search condition were also shown images of the two possible target orientations.

An example of the visual display was shown in the free-view instructions. An example of the visual display with the target emphasized was shown in the search instructions. Before each trial was a solid color display that had the average color of the beach background, followed by a blinking fixation cross subjects had to fixate on in order to trigger the trial display. Afterwards, the free-view display remained on-screen for 2 s. The search display remained on-screen for 4 s or until a response was made, whichever occurred earlier. Visual feedback stating “Correct!” or “Wrong!” in black text was given after every search trial. The experiment started with a practice block of 12 randomly selected trials from the experiment in both instruction conditions. At the end of each block, participants were allowed to take a break period.

#### Analyses

The first fixation recorded in a trial was always the fixation cross located at the center of the display, and thus removed from the analysis.

We categorized fixations based on the shortest Euclidean distance between fixations and each display item category:

1. fixations to the red turtle
2. fixations to the yellow turtle
3. fixations to the green turtle

Note that (2) and (3) will never co-occur since we manipulated the salient turtle color and only one will appear on any given trial.

(4) fixations to the purple turtles
(5) fixations on the wave

Fixations that were more than 2° visual angle away from any item were classified as a fixation on the background (accounting for our calibration accuracy threshold of 1° visual angle). Background fixations were further categorized as either the water background or the sand background. Consecutive fixations that landed near the same item, excluding background, were combined under the assumption that subject attention was on the item of interest.

It is important to note that, to compute the attentional pull towards a purple turtle, we normalized the counts of fixations towards any purple turtle and divided that count by the total number of purple turtles present on that trial.

All analysis of variance (ANOVA) results that used fixation counts as the dependent variable had group means reported as a percentage of total trials, calculated as fixations divided by the total number of fixations made in trials of the same condition. Note that red turtle orientation and background orientation were collapsed for this analysis. All ANOVA results had the Greenhouse-Geisser correction applied and degrees of freedom adjusted for violations of sphericity. T-tests results from multiple pairwise comparisons had the Bonferroni correction applied.

First fixations made within a trial in the search condition were binned into quintiles based on latency per subject and were plotted against first fixation selectivity towards the red target turtle. For each subject in the search condition, we also computed the average reaction time as a function of set size to verify the experiment setup is producing behavioral data that is consistent with visual search behavior.

### Results

#### Response times

Response times in the search condition are reported in Table 1. Response times increased logarithmically as set size increased, R^2^ = 0.89. As expected, response times were better characterized by a logarithmic trend than by a linear trend (R^2^ = 0.71). This result confirms that participants were processing the display in parallel, rejecting non-targets in an efficient manner (see Buetti et al., 2016; Lleras et al., 2020; Townsend & Ashby, 1983).

**Table 1:** Response times in search condition

Purple item set size	Average response times (ms)
0	691
2	757
4	762
8	770
16	802

#### Eye-movement data

We removed trials where there were no fixations that left the center fixation cross (2.2% of trials) or abnormal first fixation latencies (less than 50 ms or greater than 1,000 ms, which occurred in 3.0% of trials). We also removed trials with abnormal reaction times in the search condition (more than 2.5 standard deviations above or below per-subject mean reaction time in that set size condition, 0.9% of total trials) or incorrect responses in the search condition (1.3% of total trials). Altogether, 6.2% of trials were excluded.

In the search condition, on 84% of trials, subjects made a response regarding the red turtle’s orientation before they made a third fixation, ending the trial at the moment of response. Additionally, most subjects fixated on the red search target in their first fixation (43% of all search trials). This is not a concern because, as described in the *Introduction*, our hypotheses focused on analyzing first fixation selectivity for the different item categories.

#### First fixation initiation and duration times

Subjects made their first fixation much faster in the search condition (*M* = 236 ms, *SD* = 17 ms) than in the free-view condition (*M* = 352 ms, *SD* = 65 ms), *t*(21) = 7.67, *d* = 2.425, *p* <.001. Given that participants were asked to report the orientation of the turtle in the search condition, it was not surprising to find that first fixation durations on the red turtle were longer in the search condition (*M* = 591 ms, *SD* = 55 ms) than in the free-view condition (*M* = 371 ms, *SD* = 88 ms), *t*(32) = 9.43, *d =* 2.981, *p* <.001. In the search condition, first fixation durations on less-salient purple distractors were longer (*M* = 105 ms, *SD* = 18 ms) than on the salient yellow or green distractor (*M* = 94 ms, *SD* = 21 ms), *t*(19) = 3.10, *d =*.558, *p* <.05. This is consistent with a top-down account given that purple was more similar to the red in the target turtle than yellow or green.

#### Omnibus ANOVA with task instruction

We conducted an Omnibus ANOVA analysis on first fixations aimed at demonstrating that instruction conditions significantly and fundamentally altered how task instructions changed the attentional pull of the items in the scene. This ANOVA is reported for completeness purposes but, because it is not directly related to any one of the directional hypotheses described in the *Introduction*, we presented it fully in Appendix B for ease of read and clarity of presentation. The four-way ANOVA includes factors: task instruction (free-view, search) as between-subjects factor; item type (red turtle, salient turtle, purple turtle, wave, water reflection background, sand background), salient turtle color (yellow, green), and set size of purple turtles (2, 4, 8, 16) as within-subjects factors on first fixations.

**Fig. 2:**
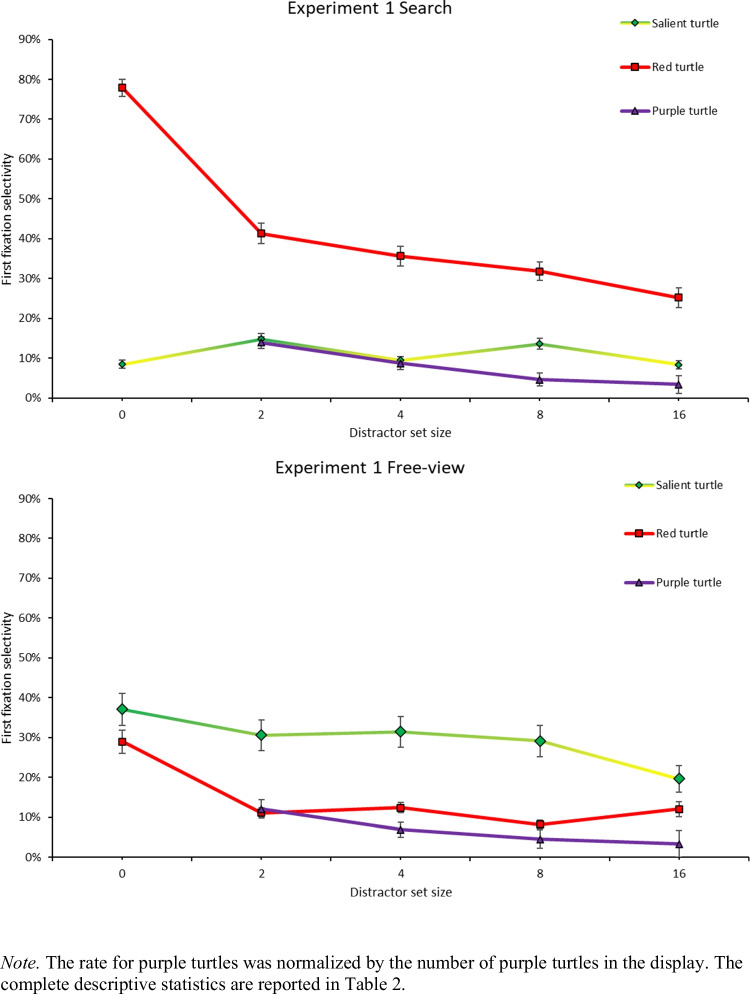
First fixation selectivity across task instructions

Overall, the results suggested that task instruction indeed modulated the looks at the different item types, as shown in Fig. 2. The salient green and yellow turtles captured more fixations in the free-view condition, validating the baseline hypothesis that bottom-up salience signals drive first fixations. The theoretical predictions described in the *Introduction* are presented more in detail below.

**Table 2:** First fixation selectivity across task conditions (s.e.)

	Free view					Search				
Set size of purple turtles	0	2	4	8	16	0	2	4	8	16
Looks at item type										
Salient turtle (yellow, green)	37% (4%)	31% (4%)	31% (4%)	29% (4%)	20% (3%)	9% (1%)	15% (1%)	9% (1%)	14% (1%)	8% (1%)
Red turtle	29% (3%)	11% (1%)	12% (1%)	8% (1%)	12% (2%)	78% (2%)	41% (3%)	36% (3%)	32% (2%)	26% (2%)
Purple turtles (group)	N/A	24% (2%)	28% (2%)	36% (2%)	53% (3%)	N/A	28% (2%)	35% (2%)	37% (2%)	55% (2%)
Purple turtle (normalized)	N/A	12%	7%	4.5%	3.3%	N/A	14%	9%	5%	3%
Wave	10% (2%)	9% (2%)	9% (2%)	9% (2%)	5% (1%)	3% (1%)	4% (1%)	5% (1%)	5% (1%)	5% (1%)
Water background	11% (2%)	12% (2%)	12% (2%)	11% (2%)	5% (1%)	5% (2%)	5% (2%)	7% (2%)	6% (2%)	3% (1%)
Sand background	13% (2%)	13% (2%)	8% (1%)	7% (1%)	5% (1%)	6% (1%)	8% (1%)	8% (1%)	6% (1%)	3% (1%)

Purple turtle (normalized) is normalized by set size after applying Equation 1. Purple turtles (group) is not normalized by set size. In free-viewing, selectivity towards turtles was unaffected by whether they were presented on a water or sand background. However, during search, turtles appearing in the water background were less likely to be fixated first than if they were in the sand background (for salient turtle, a mean decrease of 6%, *t*(20) = −5.90, *p*_*adj*_ <.001. For any purple turtle, a mean decrease of 2%, *t*(20) = −5.00, *p*_*adj*_ <.001. For the red turtle, a mean decrease of 5%, *t*(20) = −3.72, *p*_*adj*_ <.05). The asymmetrical background was implemented to keep subjects engaged during the free-viewing task, and was not relevant to our main findings. Given the small, normalized differences stemming from this asymmetry, no further analysis was done regarding the background

#### Item salience in the free-view condition

The goal of this analysis was to understand how the bottom-up characteristics of the display impacted first saccades and, more specifically, to investigate how low salient turtles impacted first fixation selectivity in the free-view condition. In addition, we wanted to validate our stimuli selection such that the yellow and green turtles were of sufficiently higher salience (through higher visual contrast) than the red (and purple) turtles and would attract fixations on a more frequent basis than any other element in the display.

**Fig. 3:**
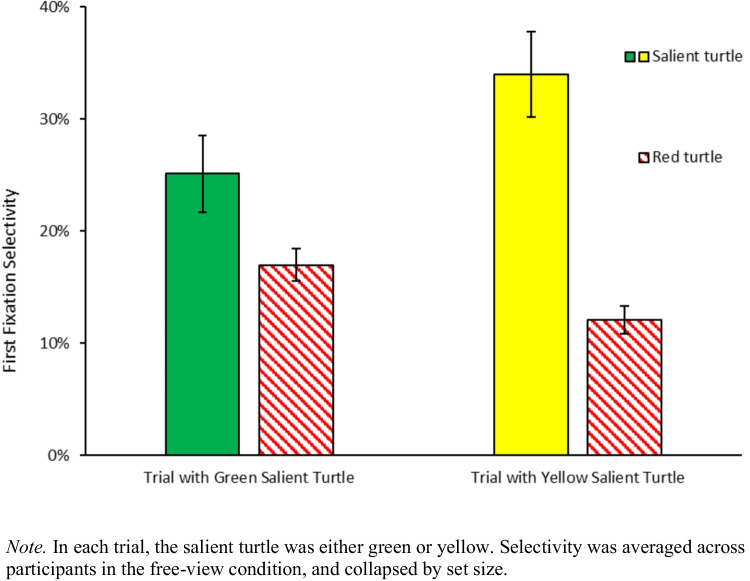
First fixation selectivity in the free-view condition

We ran a two-way ANOVA on first fixations in free-view trials (collapsed across purple turtle set sizes), with item type (red turtle, salient turtle, purple turtle, wave, water background, sand background) and salient turtle color (yellow, green) as within-subject factors. There was a main effect of item type, indicating that different items attracted the eyes to different extents, *F*(1.73, 32.92) = 20.88, ${{\eta }_{p}}^{2}$ =.524, *p*_*adj*_ <.001. First fixation rates to the salient turtle were higher (30%) than to the red turtle (15%) after Bonferroni corrections, *t*(39) = 5.19, *d =.*823, *p*_*adj*_ <.001. There was a significant interaction between the two factors, indicating that the color of the salient turtle modulated first fixation selectivity, *F*(5, 95) = 21.41, ${{\eta }_{p}}^{2}$ =.530, *p*_*adj*_ <.001. Follow-up t-tests showed that when the salient turtle was yellow, it attracted more first fixations (34%) compared to the red turtle (12%), *t*(19) = 5.51, *d =* 1.231, *p*_*adj*_ <.001; when the salient turtle was green, it attracted similar amounts of first fixations (25%) compared to the red turtle (17%), *t*(19) = 2.20, *d =*.492, *p*_*adj*_ =.60 (Fig. 3). Overall, these findings suggest that our selection of salient stimuli was effective: participants looked more at the salient turtle than towards the red turtle during free viewing.

#### Winner-take-all versus Probabilistic Salience hypotheses (free-view condition)

Given the significant three-way interaction in the OMNIBUS ANOVA above between item type, task instruction, and purple turtle set size (Appendix B), we investigated whether the number of purple turtles impacted first fixations towards the salient item. If the *Winner-take-all Hypothesis* is correct, one would not expect the number of purple turtles (low-salience items) to impact the rates of fixations directed to the more salient items in the display. If the *Probabilistic Salience Hypothesis* is correct, first fixation selectivity for the more salient items should decrease as the set size of less-salient purple turtles increases. The two-way ANOVA on first fixations for the salient turtle, with set size of purple turtles (2, 4, 8, 16) and salient turtle color (yellow vs. green) as within-subject factors showed that first fixation selectivity for the most salient item varied as a function of the number of purple turtles, as indicated by a significant main effect of set size, *F*(1.86, 35.41) = 17.46, ${{\eta }_{p}}^{2}$ =.479, *p*_*adj*_ <.001. This main effect was not qualified by salient turtle color, as indicated by the fact that the two-way interaction between purple turtle set size and salient turtle color was not significant, *F*(3, 57) = 1.24, ${{\eta }_{p}}^{2}$ =.061, *p*_*adj*_ =.30. We computed a series of follow-up contrasts to qualify this trend (Table 3).

**Table 3:** Pairwise comparisons for salient item fixations in free-view condition

Purple turtle set size (A)	Salient turtle fixations for (A)	Purple turtle set size (B)	Salient turtle fixations for (B)	*t*(39)	p_adj_
2	6.98	4	7.28	-.67	1.00
2	6.98	8	6.65	.85	1.00
2	6.98	16	4.58	5.06	*** <.001
4	7.28	8	6.65	1.67	1.00
4	7.28	16	4.58	5.54	*** <.001
8	6.65	16	4.58	4.59	*** <.001

#### Template guidance hypothesis

If the *Template Guidance Hypothesis* is correct, then fixations to the red turtle should be substantially higher in the search, template-driven condition, compared to in the free-view condition, where there is no top-down goal of attending to the red turtle. The three-way ANOVA on first fixations for the red turtle, with task instruction as between-subjects factor and set size of purple turtles (2, 4, 8, 16) and salient turtle color (green, yellow) as within-subject factors showed that first fixation selectivity for the red item is higher in the search condition (42%) than in the free-view condition (15%), as reflected by a significant main effect of task instructions, *F*(1, 38) = 72.17, ${{\eta }_{p}}^{2}$ =.655, *p*_*adj*_ <.001 (Fig. 2). Furthermore, we found a significant two-way interaction between task instruction and set size, indicating that the number of purple turtles also impacted fixation selectivity to the red turtle differently across the two task instructions, *F*(3, 114) = 17.11, ${{\eta }_{p}}^{2}$ =.311, *p*_*adj*_ <.001.

The template guidance hypothesis also predicts that purple turtles should be looked at more often in the search condition than in the free-view condition, because of their similarity to the target template. However, our results showed that any individual purple turtle was not looked at significantly more in the search condition (8%) compared to in the free-view condition (7%), *t*(317.92) = −1.30, *d =* -.145, *p*_*adj*_ =.20.

Finally, the template guidance hypothesis also predicted that, in the search condition, purple turtles should be looked at more often than yellow or green turtles, because purple is more similar to red than yellow or green. The results showed that any individual purple turtle was looked at less (8%) than the salient turtle (11%), *t*(159) = −4.63, *d =* -.366, *p*_*adj*_ <.001, indicating that bottom-up factors may have trumped top-down influences in this first fixation. We look at the interaction between bottom-up and top-down factors in more detail in the next analysis.

#### Temporal dynamics hypothesis (search condition)

**Fig. 4:**
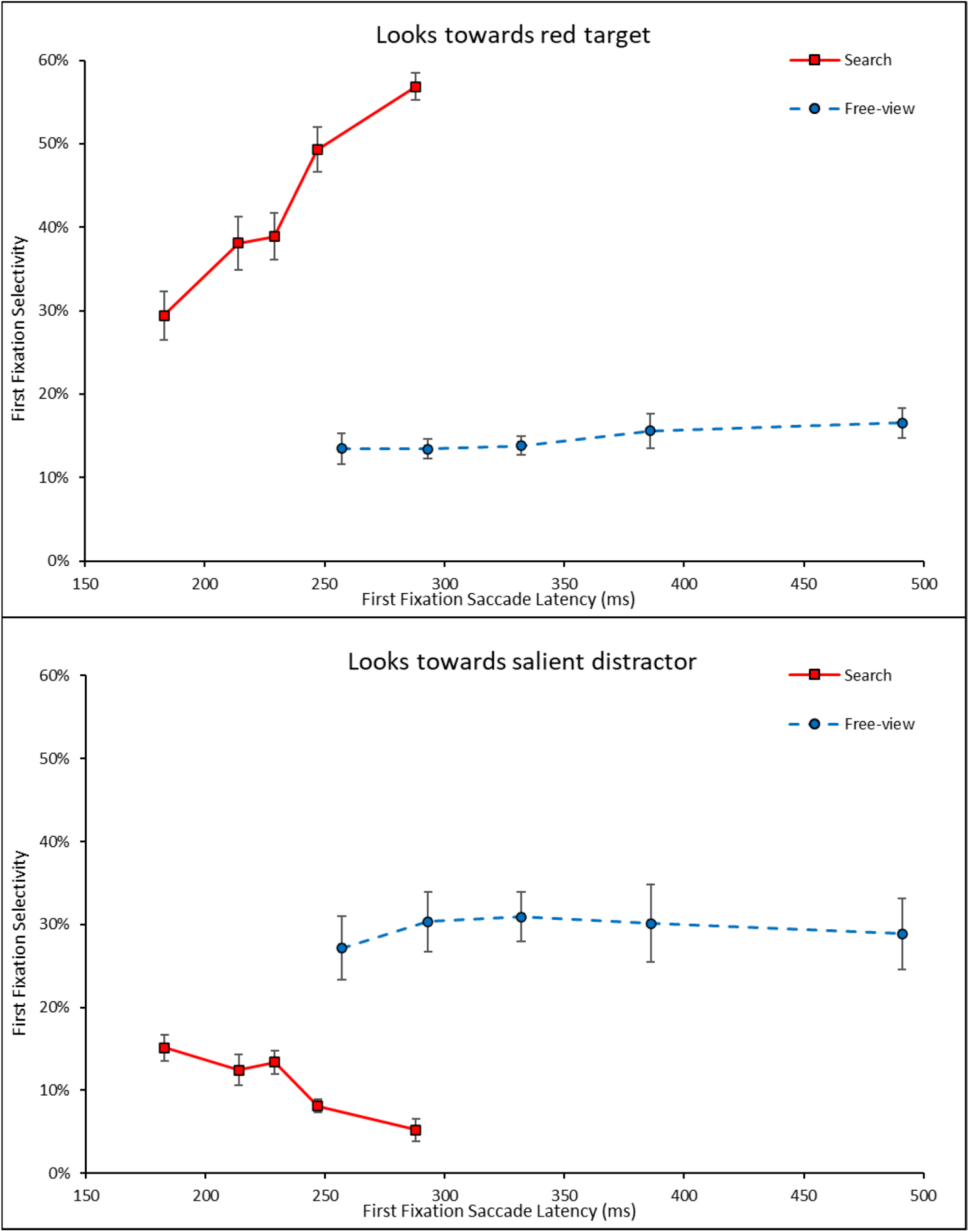
First fixation selectivity as a function of latency across task conditions

**Fig. 5:**
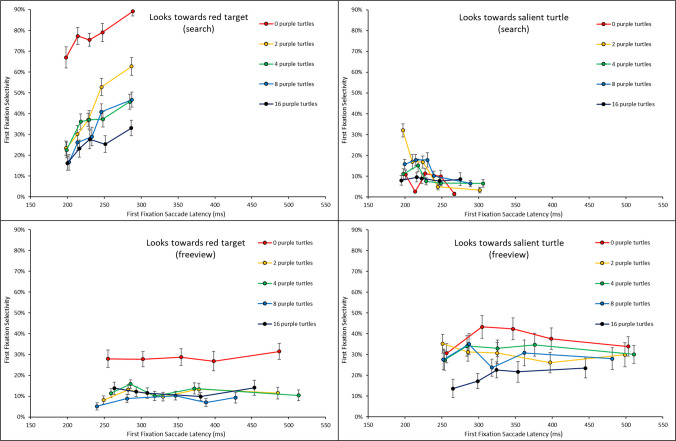
First fixation latencies by saccade latency quintiles across task conditions in Experiment 1

Following van Zoest et al. (2004), this hypothesis predicts that the fastest saccades in the search condition ought to be directed towards the salient distractor turtles more often (bottom-up pull), whereas slower saccades ought to be directed towards the red target turtle (as top-down guidance comes into play), with a transition from first to fifth quintiles between these two trends. To test this, we binned each subject’s first fixations into per-subject quintiles (Fig. 4) and ran an ANOVA with item type (red turtle, salient turtle) and saccade latency bins (5) on first fixations in the search condition. To better understand these trends, we also computed saccade latency quintiles by averaging across subjects in each task instruction condition (Fig. 5).

The significant main effect indicated that overall, first fixations landed more on the red turtle (43%) than on the salient item (11%), *F*(1, 19) = 140.83, ${{\eta }_{p}}^{2}$ =.881, *p*_*adj*_ <.001, and this was also true when considering the first quintile only (29% red versus 15% salient), *t*(19) = −3.78, *d =* -.846, *p*_*adj*_ =.001. The interaction between item type and bins was also significant, indicating that fixation selectivity to the red turtle increased across saccade latency bins, while fixation selectivity to the salient turtle decreased, *F*(2.71, 51.58) = 32.93, ${{\eta }_{p}}^{2}$ =.634, *p*_*adj*_ <.001 (Fig. 4). The upward tendency to fixate more often the red target with increasing quintiles was confirmed by the observation that first fixations landed more on the red turtle in the fifth latency quintile (57%) than in the first latency quintile (30%), *t*(19) = −9.84, *d* = −2.20, *p*_*adj*_ <.001 in the search condition. The opposite trend was true for the salient turtle, and first fixations landed less on the salient turtle in the fifth latency quintile (5%) than in the first latency quintile (15%), *t*(19) = 4.96, *d* = 1.11, *p*_*adj*_ <.001. This discrepancy in magnitudes indicates that the increase in first fixations towards the red turtle (27% increase over quintiles) cannot be fully accounted for by the decrease in first fixations towards the salient turtle (10% decrease).

By comparison, it is interesting to note that in the free-view condition, first fixation selectivity for both the salient turtle and the red turtle remained stable across bins, as evidenced by a non-significant main effect of saccade latency on first fixations in free-view, *F*(2.41, 45.84) = 1.10, ${{\eta }_{p}}^{2}$ =.055, *p* =.35. Furthermore, the three-way interaction between task instructions, item type, and saccade latency bins was also significant, *F*(2.96, 112.29) = 15.00, ${{\eta }_{p}}^{2}$ =.283, *p*_*adj*_ <.001, indicating that the pattern observed in Fig. 4 (left vs. right) were different from one another.

### Discussion

In Experiment 1, we found that in the free-view condition, the yellow or green “salient” turtle tended to attract more first fixations than the red turtle, suggesting that an item’s luminance is a bottom-up factor that attracts eye movements**.** In free view, as purple item set size increased, looks towards the salient turtle decreased. These findings give us support for the *Probabilistic Salience Hypothesis*, where we expected to see decreasing fixation selectivity for the salient turtle as we added more purple turtles. These findings also provide evidence against the *Winner-take-all Hypothesis*, wherein the properties of the less-salient items should have no effect on the fixation selectivity towards the display item with the highest attentional priority.

Turning our attention to the search condition, we found that fixation selectivity for the target decreased with increasing set size, which is consistent with findings in previous visual search studies and in line with the *Template Guidance Hypothesis*. However, per the *Template Guidance Hypothesis*, purple distractors should have drawn more first fixations than the salient distractor, but we found the opposite pattern in our results. When we normalized fixation counts for the purple turtles in the search condition, we found that the salient turtle drew more first fixations than any particular purple turtle. Moreover, purple turtles did not attract significantly more looks in the search condition than in the free-view condition. This indicates that the attentional pull of bottom-up salience (indexed here by fixations at the salient turtle) was not entirely overcome by the top-down pull of target-distractor similarity (indexed here by fixations at the purple turtles). Note that the absence of a significant difference may have also come from a lack of power for this comparison, as the frequencies tended to be numerically larger in the search condition (14%, 9%, 5%, 3%) than in the free-viewing condition (12%, 7%, 4.5%, 3.3%).

To better understand the interplay between bottom-up and top-down attentional forces, we examined the temporal dynamics of the first fixation. We found that selectivity for the red target in the search condition was lower with fastest first fixations compared to slowest first fixations. This result replicates the findings in van Zoest et al. (2004), where slower saccades were more selective towards the target in search tasks, suggesting that top-down control of eye movements requires additional processing time. With regard to fixation durations, we found that, during search, first fixation durations on the purple turtles were longer than for the green or yellow “salient” turtles. The increased duration suggests that because target-distractor color similarity was higher for the purple turtle, it may have required more time for subjects to disengage from the purple items as a non-target compared to the less-similar salient items.

One departure from van Zoest et al. (2004) in our study was that we did not find more fixations directed towards the salient turtle than to the target at the shortest saccade latencies, where bottom-up pull was expected to be the strongest. We found that the fastest saccades were still preferentially directed by the target-template because they landed more on the red turtle than on the salient turtle. van Zoest et al. (2004) suggested that these fastest saccades were more bottom-up driven, though this may instead be explained by the fact that their primary measure was the direction of the first saccade towards either the target or salient distractor, which did not allow for other characterizations of the first saccade. In the current study, items are more spaced out and fixations were able to be further categorized than just as towards the target or salient distractor. Indeed, we did find fewer fixations towards the salient distractor with longer saccade latencies, which replicates van Zoest et al. (2004); however, even at the shortest saccade latencies we found more fixations towards the target than to the salient distractor.

In sum, some general trends in our result are consistent with the results from van Zoest et al. (2004); however, one specific aspect of the results suggests a refinement to van Zoest et al.’s (2004) results is needed. Initial (fastest) saccades are not necessarily attracted to the most salient item in the display, competing for attention. These fastest saccades can successfully land on the target on a majority of trials. They also land on other items that are not the most salient (purple distractors), and the more purple distractors there are, the fewer saccades to the target there are (Fig. 5, difference between different colored lines). Thus, it is the mere presence of potential (viable) landing locations for the eyes that makes the computation of the landing location for the goal-driven saccade inaccurate. It is as if the visual system first registers the locations where there are objects in the display (a bottom-up computation), and then starts evaluating the likelihood that any one of those is the correct landing location for the eye (a top-down calculation). The impact of the salience of the signal at any one of these potential locations does not seem to be as important. This is evidenced by the fact that the number of purple distractors seem to have a clear impact on the percentage of first fixations landing on the target (most visible at the slowest quintile). When analyzing the looks at the salient item, there is a modest decrease over time of looks at the salient item, but changes in looks at this salient item do not entirely compensate for increases in looks at the target item. van Zoest et al. (2004) could not evaluate this effect that the competition for saccades is likely more driven by the *number* of objects rather than by the *salience* of these objects because their displays did not include any other good potential landing locations for the eyes: the only viable landing locations for the eyes in those displays were the target and the salient distractor (all the other elements in the display grouped to form a background texture). This finding is reminiscent of arguments made by Skow and Peterson (2005) regarding the impact of competing attention vectors in visual search: when there are several locations where subjects can move their eyes, these locations generate attention vectors that, at the moment of selection, compete with one another to determine where the eyes (or attention) eventually move. Interestingly, this competition seems to be pronounced in the presence of a time pressure and or a top-down task. Indeed, in the free-viewing condition, quintile did not have any systematic effects on fixation selectivity.

These results are also in line with the findings by Lleras et al. (2020), where it was shown that the number of “guess” saccades in efficient search tasks (i.e., saccades not initially directed at the target) is impacted by set size as well as other factors like target-distractor similarity and target eccentricity. Lleras et al. (2020) proposed that because evidence accumulation takes time, if participants decide to move their eyes prior to the end of evidence accumulation at all possible locations, the eyes will end up being directed to locations where there are items in the display, though not necessarily the target (thus the name “guess” saccades). From that perspective, what matters most is the presence of locations where evidence is being sampled from, similar to what we are arguing here.

Finally, it is worth noting that our results are somewhat different from the results typically reported in the attentional capture literature where visual features like color have been used to elicit capture. Salience arising from a featural contrast has been documented to be somewhat short-lived, arising mostly in the fastest saccades, but not necessarily present in slower ones, when irrelevant feature singletons are used in a search task (e.g., Gaspelin et al., 2016; van Heusden et al., 2022; von Mühlenen & Conci, 2016). This is in comparison to the capturing effects associated with sudden onsets, which seem to be more robust (e.g., Gaspelin et al., 2016; Theeuwes et al., 1999, Remington et al., 1992). Interestingly, here, in the free-viewing data, we observed that the attentional pull of the salient item was fairly long lived as its temporal dynamics were unaffected by time: both fast and slow first saccades were directed at the salient item at comparable rates (Figs. 4 and 5). If the feature-based salience signal dissipated over time and salience only mattered at the earliest moments of the trial (when the search items first appear), one would have predicted the tendency to look towards the most salient item to decrease over time, i.e., at the slower saccades. Thus, the salient signal, at least as indexed by the rate of spontaneous (free-viewing) saccades to the salient item, does appear to continue to be active over time. Furthermore, in the search condition (the task traditionally used to study involuntary attentional capture by salient items), we found quite modest competition for the fastest saccades coming directly from the salient item. There was only a small, overall, tendency to look at the salient item during that first saccade during search (Fig. 4) and it was about half as likely to draw the eyes as the target turtle (15% compared to 29%). In fact, the rate of target-driven saccades remained relatively low at the first quintile. Given that the fastest saccades are not necessarily directed to any specific stimulus, this result is more in line with attention being “unmoored” or in “limbo” (as proposed by van Heusden et al., 2022), from the very earliest moments in the trial, than with attention being captured by the salient item and competing against the attentional pull from the top-down target (as initially proposed by van Zoest et al., 2004). We return to this result in the *General discussion*.

## Experiment 2

In Experiment 2, we explore how top-down, target-distractor discriminability impacts eye-movement patterns in search behavior. Experiment 2 is a follow-up to Experiment 1, where we varied the color similarity between the red turtle and less-salient turtles, rather than varying the set size of the less-salient turtles. As target-distractor color similarity increases, we expected a slowdown in search efficiency (e.g., Buetti et al., 2016; Duncan & Humphreys, 1989; Lleras et al., 2020). That is, overall search performance worsens and search slopes increase. In the search condition, we expected more erroneous fixations towards less-salient distractors when their color was more similar to the target. The predictions are very similar to those for Experiment 1. Per the *Winner-take-all Hypothesis*, we would expect that in the free-view condition, fixations to the salient (green or yellow) turtle will not be impacted by the color of less-salient turtles (purple or blue). In contrast, in the *Probabilistic Salience Hypothesis*, it is possible that the color of the less-salient turtles might impact looks at the salient turtle as these less-salient turtles change the relative attentional pull to the salient turtles as a function of their own bottom-up salience. Per the *Template Guidance Hypothesis*, we expect that in the search condition, looks at the red target turtle will increase as we decrease target-distractor similarity. Additionally, looks at the salient distractor should be determined by its relative similarity to the target compared to other distractors present in the display, rather than by its bottom-up salience. Thus, distractors that are more similar to the target will draw more fixations than less similar items. We should find that in the search condition, first fixations towards the (less-salient) purple turtles should occur more frequently than fixations towards the (less-salient) blue turtles. Finally, per the *Temporal Dynamics Hypothesis*, saccades toward target should become more frequent as saccade latency increases, whereas saccades toward the salient items should become highest at the fastest saccades and decrease with increasing saccade latencies.

### Method

#### Participants

Forty-nine participants were recruited from the subject pool for Experiment 2 at the UICU in exchange for course credit or $8. Informed consent was obtained from all participants. Due to experimenter error, demographic information was not correctly collected. The recruiting process ensured that participants ranged from 18 to 52 years old, most of them undergraduate students from UIUC. Participants were excluded if data were only obtained on less than half of trials, usually as a result of poor eye tracking. Three participants in the free-view condition were excluded because they did not meet the data criterion. Participants in the search condition were excluded if their accuracy was below 85% (group mean accuracy =.99 and SDs = 0.02), or if they had mean response times more than 2.5 standard deviations away from the group average mean response time (group average mean response time = 836.09 ms, SD = 84.49 ms). One participant in the search condition was excluded because they did not meet the response time criterion. Four participants’ data contained no usable eye-tracking information due to experimenter error. Forty participants’ data were included in the analysis in Experiment 2. The final sample size was determined based on a pilot study (N = 28) that demonstrated that 40 participants were sufficient to detect the differences between the two instruction conditions in terms of first fixations to the red turtle (*d* = 1.97) and impact of low salience items on first fixations to the salient turtle in the free-viewing condition (Cohen’s *f* =.107). With α =.05 (two-tailed), a sample of 40 participants would provide >99% power to detect an effect size of *d* = 1.97 and 96% power for *f* =.107.

Apparatus, stimuli, procedure and analyses were identical to Experiment 1, except where indicated.

#### Stimuli

The stimuli were the same as in Experiment 1 except we added low-similarity blue turtles, which were defined at HSL values of (196, 60%, 24%) The corresponding average RGB values for blue were (24, 78, 98).

#### Design

Subjects were randomly assigned to an instruction condition as in Experiment 1. The red and salient turtle were always present along with eight less-salient turtles. There were two less-salient turtle colors (blue, purple) and two salient turtle colors (yellow, green). Red turtle orientation (facing left, right) and background orientation (water reflection pattern on left, right) were controlled for, resulting in 16 conditions (2 × 2 × 2 × 2) that were repeated six times, summing up to a total of 96 trials. The trials were presented in a block design, where all 16 conditions were presented once in a block and in random order across subjects. For analysis, the red turtle orientation and background orientation were collapsed.

#### Analyses

The analyses performed were the same as in Experiment 1 except we added a fixation destination category for blue turtles. Note that blue and purple turtles never co-occur and only one type of less-salient turtles appeared on any given trial.

### Results

#### Response times

Response times in the search condition with the higher-similarity purple turtles (*M* = 855.90 ms, *SD* = 84.09 ms) were longer than on trials with lower-similarity blue turtles (*M* = 789.16 ms, *SD* = 78.55 ms), *t*(38) = 2.59, *d =*.820, *p* <.05, confirming the effectiveness of the target-distractor similarity manipulation.

#### Eye-movement data

We removed trials where there were no fixations that left the center fixation cross (3.6% of trials) or abnormal first fixation latencies (less than 50 ms or greater than 1,000 ms, 1.7% of trials). We also removed trials with abnormal reaction times in the search condition (more than 2.5 standard deviations above or below per-subject mean reaction time in that distractor-similarity condition, 1.0% of total trials) or incorrect responses in the search condition (0.5% of total trials). Altogether, 6.8% of trials were excluded.

In 81% of trials in the search condition, subjects made a response regarding the red turtle’s orientation before they made a third fixation, ending the trial at the moment of response. Additionally, many subjects fixated on the red search target in their first fixation (33% of all trials).

#### First fixation initiation and duration times

As in Experiment 1, subjects made their first fixation earlier in the search condition (*M* = 245 ms, *SD* = 25 ms) compared to the free-view condition (*M* = 355 ms, *SD* = 79 ms), *t*(23) = 5.84, *d* = 1.846, *p* <.001. Similarly, first fixation durations on the red turtle were longer in the search condition (*M* = 596 ms, *SD* = 105 ms) than in the free-view condition (*M* = 387 ms, *SD* = 171 ms), *t*(32) = 4.64, *d =* 1.467, *p* <.001. In the search condition, first fixation durations were longer on less-salient turtles (*M* = 104 ms, *SD* = 22 ms) than on salient turtles (*M* = 83 ms, *SD* = 22 ms), *t*(19) = 3.49, *d =*.951, *p* <.05. That said, there was no significant difference in first fixation durations between purple distractors (*M* = 103 ms, *SD* = 19 ms) and blue distractors (*M* = 105 ms, *SD* = 39 ms), *t*(19) = -.21, *d =*.049, *p* =.84.

#### Omnibus ANOVA with task instruction

**Fig. 6:**
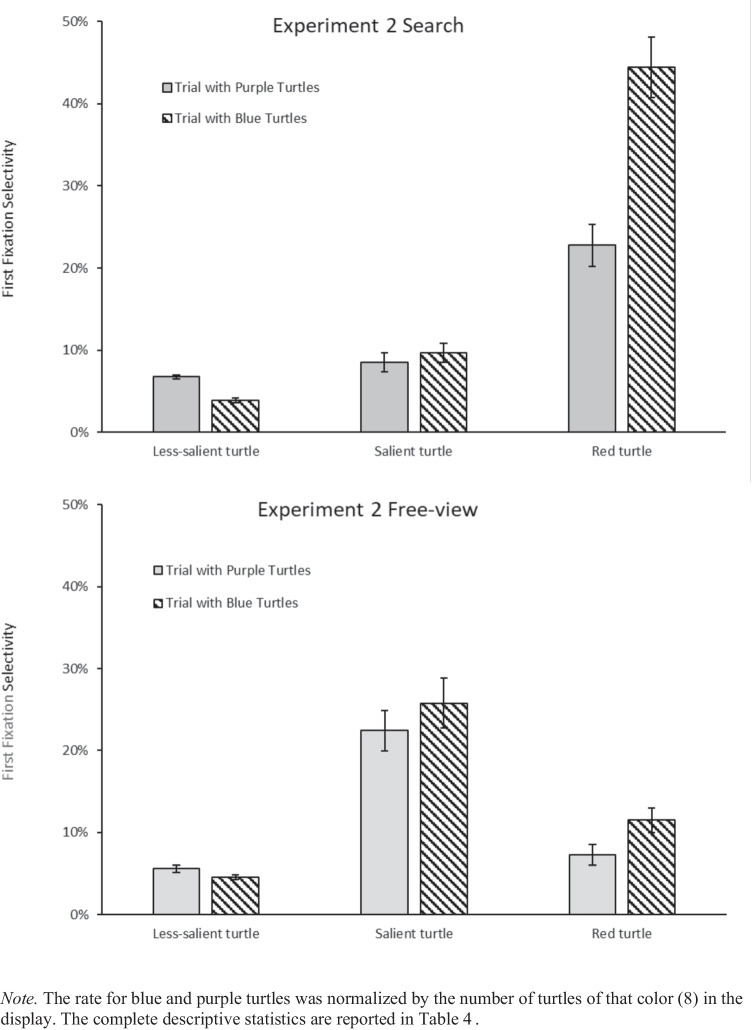
First fixation selectivity across task instructions.

As with Experiment 1, we ran a four-way ANOVA, with task instruction (free-view, search) as between-subjects factor, and item type (red turtle, salient turtle, less-salient turtle, wave, water reflection background, sand background), salient turtle color (yellow, green), and less-salient turtle color (purple, blue) as within-subject factors on first fixations. The results of this ANOVA are presented in Appendix C. As in Experiment 1, task instruction significantly modulated the looks at the different item types (see Fig. 6).

**Table 4:** First fixation selectivity across task conditions (s.e.)

Looks at item type	Free view		Search	
	Color of less-salient turtles		Color of less-salient turtles	
	Purple	Blue	Purple	Blue
Salient turtle (yellow, green)	22% (2%)	26% (3%)	8% (1%)	10% (1%)
Red turtle	7% (1%)	12% (2%)	23% (3%)	44% (4%)
Less-salient turtles (group)	45% (3%)	37% (2%)	54% (2%)	31% (2%)
Less-salient turtle (normalized)	5.6%	4.6%	6.8%	3.9%
Wave	10% (3%)	12% (3%)	4% (1%)	4% (1%)
Water background	8% (1%)	7% (1%)	5% (1%)	6% (1%)
Sand background	8% (1%)	7% (1%)	6% (1%)	6% (1%)

#### Item salience in the free-view condition

Again, the goal of the analysis was to understand how the bottom-up characteristics of the display impacted first saccades and, more specifically, to investigate how low-salient turtles impacted first fixation selectivity in the free-view condition. This analysis also allowed us to verify that the turtles designed to be less salient (purple and blue) were indeed capturing fewer fixations than those designed to be more salient (yellow and green).

**Fig. 7:**
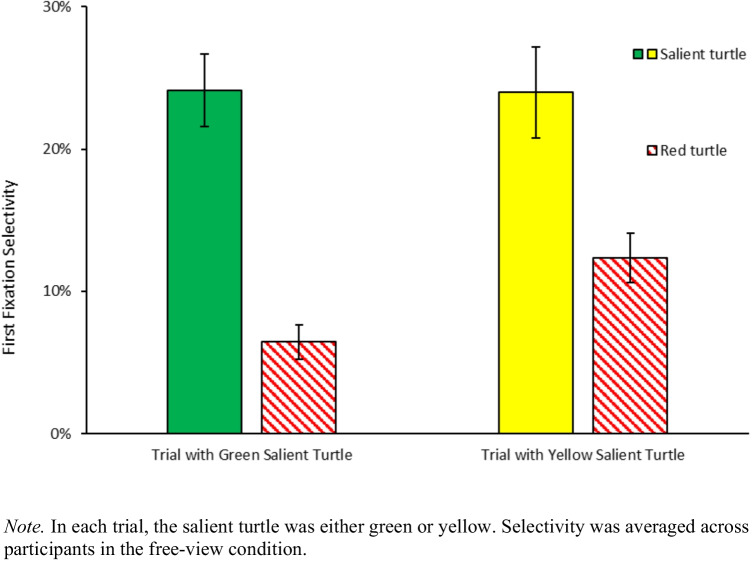
First fixation selectivity in the free-view condition

We ran a three-way ANOVA on first fixations in free-view trials with item type (red turtle, salient turtle, less-salient turtle, wave, water background, sand background), less-salient turtle color (purple, blue), and salient turtle color (yellow, green) as within-subject factors. There was a main effect of item type, indicating that different items attracted the eyes to different extents, *F*(2.53, 47.98) = 40.37, ${{\eta }_{p}}^{2}$ =.680, *p*_*adj*_ <.001. First fixation rates to the salient turtle were higher (24%) than to the red turtle (9%) after Bonferroni corrections, *t*(79) = 8.98, *d =* 1.004, *p*_*adj*_ <.001. There was a significant interaction between item type and salient item color, indicating that the color of the salient turtle modulated first fixation selectivity, *F*(2.85, 54.24) = 7.45, ${{\eta }_{p}}^{2}$ =.282, *p*_*adj*_ <.001. Follow-up t-tests showed that when the salient turtle was yellow, it attracted more first fixations (24%) compared to the red turtle (12%), *t*(39) = 4.11, *d =*.649, *p*_*adj*_ <.05; when the salient turtle was green, it attracted more first fixations (24%) compared to the red turtle (6%), *t*(39) = 12.35, *d =* 1.952, *p*_*adj*_ <.001 (Fig. 7). There was also a significant interaction between item type and less-salient turtle color, indicating that the color of the less-salient turtle modulated first fixation selectivity, *F*(2.7, 51.29) = 9.76, ${{\eta }_{p}}^{2}$ =.339, *p*_*adj*_ <.001. However, the three-way interaction was not significant, *F*(3.04, 57.77) =.32, ${{\eta }_{p}}^{2}$ =.017, *p*_*adj*_ =.81, and less-salient item color did not seem to modulate the effect of the salient item color on first fixations. Overall, these findings suggest that our selection of salient stimuli were effective: participants looked more at the salient turtle than towards the red, purple and blue turtles during free viewing.

#### Winner-take-all versus Probabilistic Salience Hypotheses (free-view condition)

We ran a two-way ANOVA on first fixations for the salient item in the free-view condition, with less-salient turtle color (purple and blue) and salient turtle color (yellow and green) as within-subject factors. We found a significant main effect of less-salient turtle color on first fixations towards the salient turtle, *F*(1, 19) = 7.20, ${{\eta }_{p}}^{2}$ =.275, *p*_*adj*_ <.05. A follow-up t-test revealed that there were more first fixations towards the salient turtle when the less-salient turtles were blue (26%) than when they were purple (22%), *t*(39) = 2.62, *d =*.414, *p*_*adj*_ <.05. This main effect was not qualified by salient turtle color, as indicated by the fact that the two-way interaction between less-salient turtle color and salient turtle color was not significant, *F*(1, 19) =.64, ${{\eta }_{p}}^{2}$ =.032, *p*_*adj*_ =.44. These findings support the *Probabilistic Salience Hypothesis* because the information on the low-salience end of the spectrum impacts the selectivity for the highest salience item in the scene, even when controlling for the number of objects in the display.

#### Template guidance hypothesis

First, we looked at the fixations to the red turtle which were expected to be substantially higher in the search condition compared to the free-view condition. The three-way ANOVA on first fixations for the red turtle, with task instruction (search, free-view) as between-subjects factor, and less-salient turtle color (purple, blue) and salient turtle color (green, yellow) as within-subject factors showed that first fixation selectivity for the red item is higher in the search condition (34%) than in the free-view condition (9%), as reflected by a significant main effect of task instructions, *F*(1, 38) = 56.21, ${{\eta }_{p}}^{2}$ =.597, *p*_*adj*_ <.001 (Fig. 7).

Second, the looks at the red target turtle should decrease as we increase target-distractor similarity in the search condition. This was confirmed by the result showing more looks for the red turtle when the less-salient turtles were less similar (blue, 44% for red) than when they were more similar (purple, 23% for red), *t*(39) = 10.41, *d =* 1.646, *p*_*adj*_ <.001.

Third*,* first fixations towards the less-salient purple turtles in the search condition should occur more frequently than fixations towards the blue turtles because purple is more similar to the target color (red) than blue (by design). The results confirmed more looks were directed at less-salient turtles when they were purple (7%) than when they were blue (4%), *t*(39) = −9.49, *d =* −1.501, *p*_*adj*_ <.001.

Fourth, looks at purple turtles should also be more frequent in the search condition than in the free-view condition because in the search condition they are the turtles most similar to the target, whereas in the free-view condition they are some of the least salient turtles in the scene. This was confirmed by the results: we found more looks for any particular purple turtle in the search condition (7%) than in the free-view condition (6%), *t*(67.9) = −2.50, *d =* -.558, *p*_*adj*_ <.05. In contrast, when we decrease target-distractor color similarity, we found that looks for any particular blue turtle in the search condition (5%) was not significantly different than in the free-view condition (4%), *t*(75.8) = 1.44, *d =*.323, *p*_*adj*_ =.15.

#### Temporal dynamics hypothesis

**Fig. 8:**
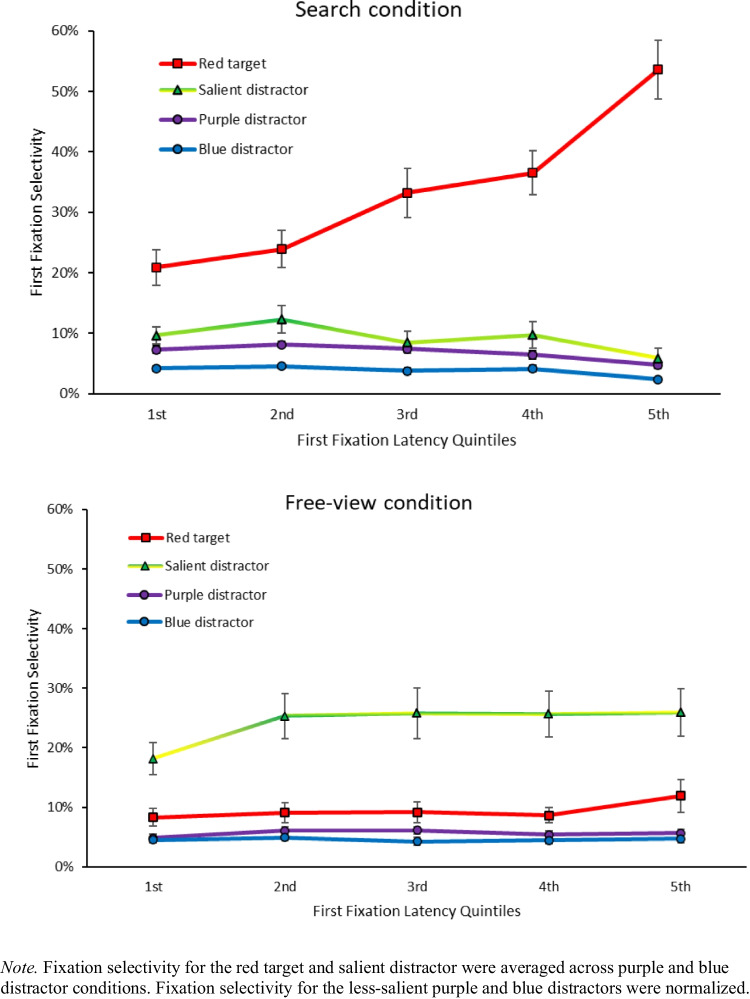
First fixation selectivity as a function of latency across task conditions

As in Experiment 1, we tested how the selectivity of the first fixation dynamically varied as a function of saccade latency by conducting an ANOVA with item type (red turtle, salient turtle) and saccade latency bins (5) on first fixations in the search condition. The significant main effect indicated that in search, first fixations landed more on the red turtle (34%) than on the salient turtle (9%), *F*(1, 19) = 56.58, ${{\eta }_{p}}^{2}$ =.749, *p*_*adj*_ <.001, and this was also true when considering the first quintile only (21% red vs. 10% salient), *t*(19) = −3.44, *d =* -.769, *p*_*adj*_ <.05. The interaction between item type and saccade latency bins was also significant, indicating that fixation selectivity to the red turtle increased across bins, while fixation selectivity to the salient turtle remained stable, *F*(4, 76) = 18.07, ${{\eta }_{p}}^{2}$ =.487, *p*_*adj*_ <.001 (Fig. 8, left). This was confirmed by comparing first fixations to the red turtle in the first (29%) versus the fifth quintile (57%), *t*(19) = −10.92, *d* = −1.68, *p*_*adj*_ <.001, showing a significant increase with saccadic latency. In contrast to Experiment 1, first fixations to the salient turtle between the first (10%) and fifth quintiles (6%) were not significantly different, *t*(19) = 2.37, *d* =.53, *p*_*adj*_ =.28.

We also looked at the impact of target-distractor similarity on first fixations towards less-salient turtles (purple, blue) as a function of saccade latency bins (5) in the search condition. The ANOVA with less-salient item color (purple and blue) and saccade latency bins (5) showed that first fixations landed more on a purple turtle (7%) than on a blue turtle (4%), *F*(1, 19) = 75.70, ${{\eta }_{p}}^{2}$ =.799, *p*_*adj*_ <.001, leading to a main effect of less-salient item color. We also found a significant main effect of bins, *F*(4, 76) = 10.79, ${{\eta }_{p}}^{2}$ =.362, *p*_*adj*_ <.001, indicating that at the slowest saccades, looks towards less-salient distractors decreased. The interaction between less-salient turtle color and bins was not significant, indicating that looks towards the less-salient distractors decreased across bins, regardless of their color, *F*(4, 76) = 2.25, ${{\eta }_{p}}^{2}$ =.106, *p*_*adj*_ =.71 (Fig. 8), or that we did not have enough power to detect any differences between these two conditions.

Finally, as also observed in Experiment 1 in the free-view condition, first fixation selectivity in free-view for both the salient turtle and the red turtle remained stable across bins, as evidenced by a non-significant main effect of saccade latency on first fixations in free-view, *F*(4, 76) = 1.80, ${{\eta }_{p}}^{2}$ =.087, *p* =.13. The three-way interaction between task instructions, item type, and saccade latency bins was also significant, *F*(4, 152) = 10.53, ${{\eta }_{p}}^{2}$ =.217, *p*_*adj*_ <.001, indicating that the patterns observed in Fig. 8 (left vs. right) were different from one another.

### Discussion

With respect to the *Winner-take-all Hypothesis,* we replicated the findings from Experiment 1 in Experiment 2, where in the free-view condition, first fixation selectivity for the salient turtle changed with respect to properties of the less-salient turtles. These findings give us support for the *Probabilistic Salience Hypothesis*, where we expected to see changes to fixation selectivity as we change the color of the less-salient turtles.

We found that fixation selectivity for the target decreased with increasing target-distractor similarity, which is consistent with findings in previous visual search studies and again in line with the *Template Guidance Hypothesis*. Also, the high-similarity purple distractors attracted more first fixations than the low-similarity blue distractors, again giving support to the *Template Guidance Hypothesis.* In contrast with Experiment 1, first fixations to purple turtles did occur more frequently in the search condition than in the free-view condition.

Furthermore, in search, first fixation durations on the more-similar less-salient turtles were longer than for the less-similar salient turtles, again in agreement with the Template Guidance hypothesis. However, we did not find a significant difference in first fixation durations between the blue and purple less-salient distractors, indicating that either disengagement occurred at a comparable speed for the two levels of turtle similarity to the target, or that our methods were not sensitive enough to reliably measure such differences. Alternatively, it may be that, in addition to top-down similarity, bottom-up factors may also contribute to determine disengagement times during search.

Finally, with regards to first fixation timings, selectivity for the red target in the search condition was lower at the fastest initial saccades compared to slowest initial saccades, replicating Experiment 1 and the findings in van Zoest et al. (2004). However, and as in Experiment 1, fixations to the salient turtle were once again relatively few and did not decrease to the extent that fixations to the target increased as quintiles increased. It is once again suggestive that the reason fast fixations do not go to the target is not because of salience-driven competition but most likely because of location competition associated with other possible landing locations for the eyes (i.e., less salient items).

## General discussion

In this study we investigated the contributions of top-down and bottom-up processes towards eye movements in visual search and during free viewing. With regard to bottom-up signals, we utilized controlled stimuli to provide estimates of bottom-up salience signals in our displays. In free-view conditions, Experiment 1 results showed first fixation selectivity towards the most salient item decreased as set size increased. Similarly, Experiment 2 results showed selectivity towards the most salient item varied as a function of the color of less salient items.

These sets of results are strongly supportive of bottom-up attentional capture being a relative rather than absolute effect: it is not the case that the most salient item in a display (once selected) is processed independently of other items in the display, in a winner-take-all fashion. Rather, the degree to which it captures attention depends on the other attentional forces in the display, very much in line with what is proposed by the Normalization Theory of Attention, where any one element has an attentional pull, the strength of which is partly determined by its visual characteristics and partly determined by the visual characteristics of all other items in the display (via normalization). The salient item does not get processed in isolation – its attentional pull is modulated by the broader visual context.

**Fig. 9:**
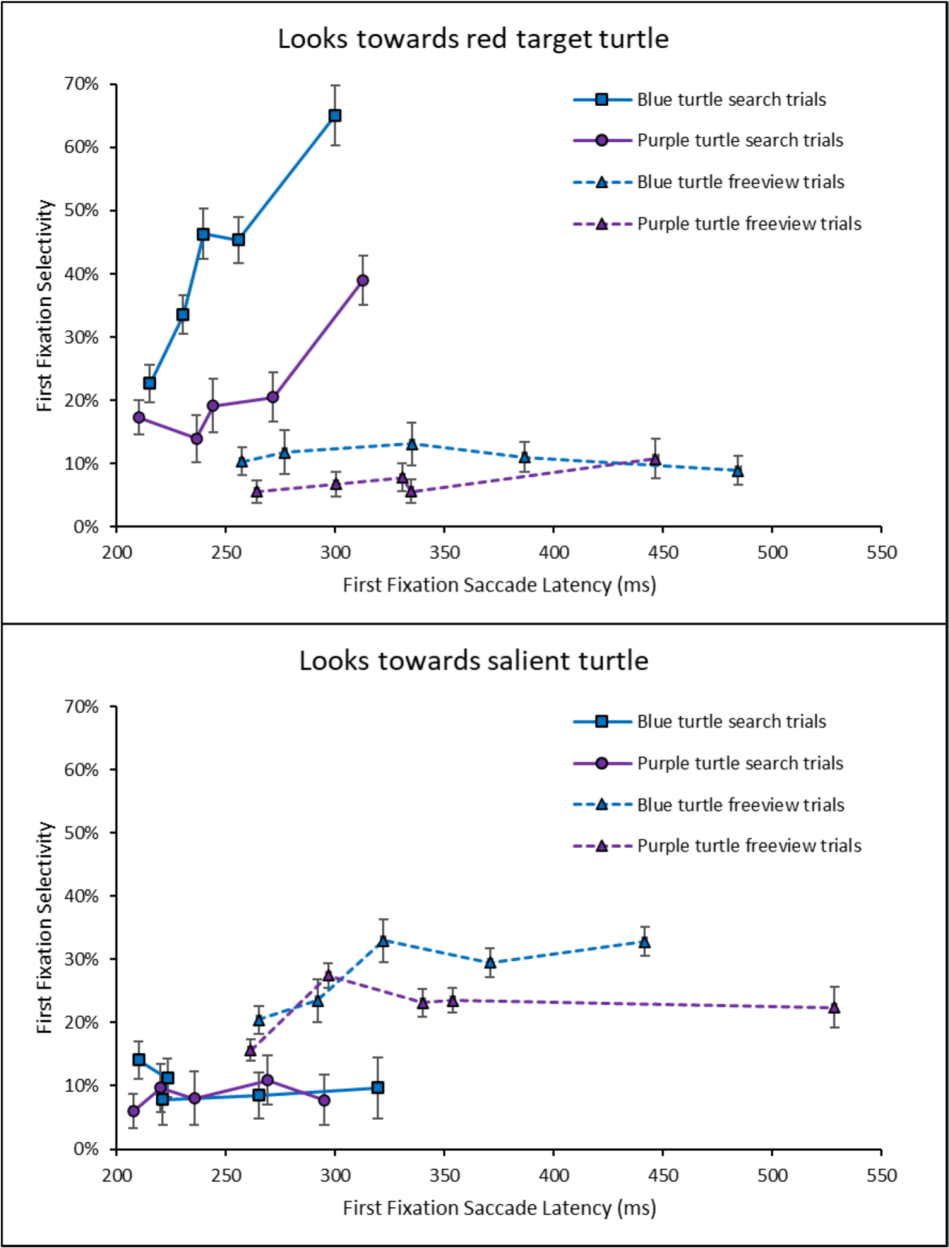
Saccade latency quintiles across task conditions

Liesefeld et al. (2016) proposed target saliency as the primary driver of visual search behavior, suggesting that differences in search performance may be explained purely by quantitative differences in target discriminability. However, our results showed that bottom-up factors cannot determine or explain search behavior by themselves. Indeed, in free viewing, participants clearly tended to first look at the salient item the most, across both experiments. In other words, the discriminability of the target turtle, as evaluated when bottom-up factors can most systematically impact performance, does not predict fixations during search. Similarly, we can conclude that the bottom-up signal of the most salient item in the display plays little to no role in impacting search behavior, or at least that first saccade during search: first saccades to the most salient turtle are relatively few. They also only tend to be observed at the fastest saccades. Intermediate or longer first saccades tend to rarely go towards the most salient turtle. In contrast, what seems to be the most important determinant of eye behavior are two well-known factors in visual search: set size (i.e., total number of elements that can potentially be the target) and the similarity relationship that these elements have with respect to the target. For instance, it is substantially easier to direct an initial saccade towards the target when it is embedded around blue turtles – low similarity distractors – than when it is presented among purple turtles – the high similarity distractors (see Experiment 2, Fig. 9). This is in spite of the fact that blue and purple turtles do not tend to elicit bottom-up capture to substantially different degrees (see Fig. 8, Table 4). There is a 5.6% versus 4.6% rate in initial fixations to individual purple and blue turtles, respectively, in free viewing. This represents only a 21% higher rate for purple than blue turtles. In contrast, during search, this difference grows to 6.8% versus 3.9%, that is, there is a 74% higher rate for first fixations to purple than blue turtles.

These results align well with the current Target Contrast Signal Theory (Lleras et al., 2020), where it was proposed that the main factor determining search performance in efficient search (with a fixed target) was the magnitude of the featural contrast between the target and distractor items: the larger the contrast, the easier it is to find the target. Note that this target contrast is computed in the participants’ head and it is not easily nor bottom-up computable in the display. This is because target and distractor stimuli can be separated by large regions of the display and this relationship still holds. The relationship also holds in very sparsely populated displays (like in small set size conditions in Experiment 1). Thus, it cannot be argued that the bottom-up signal (which reflects a local contrast of features between an item and its immediately surrounding region) is the same as, or can incorporate the same information as, the top-down contrast.

Perhaps the most significant new result from the current study relates to the temporal dynamics analysis of the first fixation. In the search condition, the data show that goal-driven saccades take time to be programmed correctly (i.e., to find the correct landing target). Initial saccades that are executed relatively early are, therefore, ill-informed and may miss the target at relatively large frequencies. As time unfolds, the visual system is better able to figure out which of the potential landing locations in the display contains the target, and this is reflected in a steady increase of initial saccades landing directly on the target. Surprisingly, it is not the case that the fastest saccades look “bottom-up driven,” as had been proposed by van Zoest et al.’s classic work (see also van Heusden et al., 2022). Or at least not bottom-up in the traditional sense: even the fastest first saccades (first quintile) are not directed towards the most salient turtle at the rate that they are fixated during free-viewing. Furthermore, the number of elements in the display appears to have a much more dramatic effect on performance (Fig. 5 top left panel) than the potential capturing effect of a single bottom-up salient item (Fig. 5 top right panel). The impact of number of locations is pronounced at all latencies, and indeed is most pronounced at the slowest latencies (i.e., differences between color lines in Fig. 5 top left panel), whereas the impact of the singleton diminishes as saccadic latencies get longer (color lines collapse and approach zero in Fig. 5 top right panel).

In sum, our results provide empirical support for van Heusden et al.’s (2022) theoretical framework regarding the role of saliency in visual search. van Heusden et al. (2022) proposed that saliency’s primary function is to identify potential target locations rather than directly drive selection. Our data extends this framework by demonstrating how this plays out in both search and free-viewing conditions: what makes the fastest saccades inaccurate during search is not primarily competition from salient objects (the traditional “singleton” effect in the attentional capture literature), but rather the presence of multiple potential landing locations that must be evaluated. Yes, the presence of these locations is established via bottom-up analysis of the scene, but it is their presence, more so than their bottom-up conspicuity, that impacts these fast saccades. The more there are, the more complex the decision process is, simply because there are more possibilities to evaluate. And, as time unfolds, each of these possible locations begins to be evaluated, in a top-down fashion, to determine the location containing the target. This explains why top-down effects like set size (Fig. 5) and target-distractor similarity (Fig. 9) become more pronounced over time (i.e., at later saccade latencies).

The interpretation of the fastest saccades being ill-informed is very much in line with the concept of attentional limbo, whereby saccadic selectivity is not affected by relative salience, nor by an item’s top-down relevance (van Heusden et al., 2022). That said, one difference between our results and those in van Heusden et al. (2022) is that those authors found evidence for attentional limbo at a moment after attention had been captured by bottom-up salience and before attention being driven by top-down control. (Their empirical data showed that salience signals had a larger influence when saccades had latencies of up to about 240 ms, followed by a brief attentional limbo (non-selectivity), followed by top-down control.) We found little evidence for that early, bottom-up capture, instead finding evidence for attentional limbo at the fastest saccades, before attention began responding to top-down control signals (the attentional sequence was: attentional limbo, followed by top-down control). Our data showed that three of the five quintiles of saccadic latencies are within that < 240 ms timeframe, which is in line with the saliency-driven timeframe found in van Heusden et al. (2022), yet the goal-relevant target was still the most selected item (and not the salient item). This suggests that either the bottom-up capture signal had already been over-ridden (perhaps as proposed by the signal suppression theory of Gaspelin & Luck, 2017) or that perhaps it was never there to begin with. More research is needed to sort out these two possibilities. Indeed, van Heusden et al. (2022) proposed that the time courses of the bottom-up and top-down signals are independent. Thus, one should not necessarily predict that as the bottom-up one falls, the top-down one rises. If the two temporal signals are indeed independent, it may be possible that the bottom-up is not even observed to begin with (or only minimally so), while the other one only strengthens over time.

### Relation to FVF (Functional Visual Field) theory of visual search

Hulleman and Olivers (2017) posited that visual search behavior can be modeled solely by eye movements and the information contained within functional viewing fields, obsoleting the need to account for properties of individual items. In this study, stimuli were constructed such that most of the information needed for visual search could be obtained in under three fixations, and yet we find that the number of items in the display was a critical determinant of behavior: it produced a logarithmic increase in search response times (Experiment 1) and was a critical factor in determining *where* the eyes would land, and more specifically, in determining the likelihood that the target would be fixated in the first fixation. We believe a best approach might be to marry the idea that the eyes capture sub-regions of the display at a time (FVF-driven search), with the idea that, during any one fixation, item analysis rules the pre-eminent role in determining behaviors (like future fixations, fixation duration, total response time).

### Relation to attentional capture

The visual distinctiveness of an item in a scene can arise from computations occurring at different levels of the visual processing hierarchy. Previous work investigating attentional capture have often used the term salience to refer to the visual uniqueness of the singleton item in a display. In most attentional capture studies, feature-based salience differences between stimuli are relatively small or non-existent. Every item in the display (typically letters or squares) are fully saturated, colored stimuli that produce a large contrast signal against their immediate background (most often, a black background surface). Thus, as far as early/low-level vision is concerned, such stimuli are probably all equally salient in low-level terms and equally likely to capture attention. It appears then that the property of the stimulus that triggers attentional capture in attentional capture studies arises from the mid- or higher-level vision computations, determining the uniqueness of the singleton stimulus. When visual representations become more complex, one of the items can become more “distinct” by virtue of being different from the others. This requires the visual system to be able to group and compare elements to be able to ascertain this visual distinctiveness from the other items in the display. In other words, there exists a level of representation in the visual system that can be used to determine that one of the items is relatively “unique” compared to the others (perhaps utilizing perceptual grouping processes). This is not too different from what Theeuwes (2023) recently argued to explain why shape-homogeneous attentional capture displays are more likely to produce an attentional capture effect by a color singleton, than shape-heterogeneous displays.

We believe it is important to appreciate the difference between the concept of *uniqueness,* as captured by a mid-level or high-level comparison of visual representations, and that of *salience*, as reflected by low-level or early vision local contrasts. Salience (also referred to as saliency in the initial work by Itti & Koch, 2000) reflects the total magnitude of local contrast of an item against its background, along a sum of different visual dimensions (e.g., color, luminance, orientation). We think it would be useful for the literature to start distinguishing the terms salience and uniqueness. Indeed, an object in a display can be salient, unique, or both. A recent paper by Kotseruba et al. (2020) elegantly made this point. It demonstrated that high-performing salience toolboxes (developed to respond to the low-level features that drive the eyes, i.e., to respond to *salience*), while able to predict quite well fixations in free-viewing tasks, are unable to predict human performance in “uniqueness” detection tasks (oddball tasks with either geometric shapes or realistic oddball images). This is presumably because these toolboxes do not represent the mid- to high-level properties required to compute uniqueness (like humans do). As a result, these salience toolboxes are unable to predict fixations towards a unique element in a scene, if that element is of comparable low-level salience to all the other objects in the scene. This is a finding that was present already in the very first saliency toolbox (Itti & Koch, 2000).

In the present study, we designed our stimuli to vary in terms of salience: turtles were defined to differ in terms of their luminance and color contrasts with regard to the sand background. The salient turtles were more salient in terms of their low-level properties than the red and purple turtles. We also created relatively sparse displays, to minimize the likelihood that the visual features of one turtle would impact the contrast of nearby turtles. Perhaps this aspect of our design explains why we find that salient signals are sustained over time in the free-viewing conditions, when other researchers would have predicted a fading impact of salience on fixation selectivity over time (e.g., Gaspelin et al., 2016; van Heusden et al., 2022; von Mühlenen & Conci, 2016). It might also explain why the salient signal might be more easily suppressed in the search task condition: an early arising signal, like salience, may have more opportunities to be silenced early in time than a relatively later arising signal, like uniqueness. Thus, salience might drive eye movements in free viewing, as we observed here, because it is a stable signal that has no reason to be suppressed under this instruction.

### Free-viewing interpretations

Henderson et al. (2018) have shown that eye movements in free-viewing contexts may be driven more by meaning and semantics rather than low-level salience. One possible interpretation of our results in the free-viewing condition is that subjects’ eye movements were being guided by meaning, and so those eye movements cannot act as a baseline measure of bottom-up saliency. We note, however, that our stimuli differ from those used in Henderson et al. (2018), as well as other studies that utilize real world scenes as stimuli. They find effects of meaning when using real world scenes with varied levels of semantic meaning, often with different objects and backgrounds. In our study, we have manipulated only one type of object (pseudorealistic turtles). We believe it is unlikely that the semantic meaning of these turtles varied much as a function of the low-level visual properties we manipulated. Furthermore, we manipulated physical properties of the turtle stimuli that should affect low-level salience, like color contrast and luminance contrast with the background. In other words, we manipulated properties of the stimuli that are known to a priori impact low-level salience of these stimuli. We also confirmed the success of this assumption, by measuring the salience of our various turtles using the GBVS saliency toolbox (Harel et al., 2007). The analysis is presented in Appendix A. Finally, the constant background of the beach, although it has idiosyncrasies like the middle wave and water reflection pattern, did not vary across trials, and thus is unlikely to have affected eye movements through semantic guidance, or salience, for that matter (e.g., Gaspelin et al., 2016; Wang & Theeuwes, 2018). Thus, we find it overwhelmingly more likely that the eye-movement patterns in free viewing we have found were due to the effect of salience, rather than meaning or scene semantics.

Another issue to consider when establishing the bottom-up baseline in the free-viewing condition is that subjects may have self-imposed or inferred a top-down goal that we as researchers were interested in. However, this is unlikely given the results, because if the free-viewing eye-movement patterns were top-down driven like in the search condition, then subjects should have displayed the same signature pattern that we observed in the search condition, where eye movements become more precise (in terms of achieving their self-imposed goal or looking at the item of top-down interest) as time passes during the trial. There does not seem to be any evidence of an incoming top-down influence on eye movements, whereby participants become more likely to look at the salient item as trial time passes. A quick glance at Fig. 5 illustrates this: first fixations towards the items during free viewing did not meaningfully change as a function of trial time. Additionally, saccades were substantially slower in the free-viewing condition compared to the search condition, indicating that participants were not attempting to quickly produce a behavior consistent with a time-sensitive internal goal.

### Limitations in generalizability

Our study’s generalizability is limited by its focus on efficient, parallel visual search, which may not represent real-world search scenarios. Many search trials in our study concluded during the participants’ first fixation due to the spaced-out nature of our stimuli. This design allowed for efficient search using peripheral vision but limited our ability to analyze how fixations evolve over time in more complex search tasks. While we used more complex stimuli and backgrounds than typical visual search experiments, we avoided item clusters, which are common in real-life environments and often drive eye movements as people attempt to see better what is at crowded locations. The spaced-out nature of our stimuli, while allowing for efficient parallel search, might not extend to search situations that demand a more serial search strategy.

## Figures and Tables

**Fig. 10:**
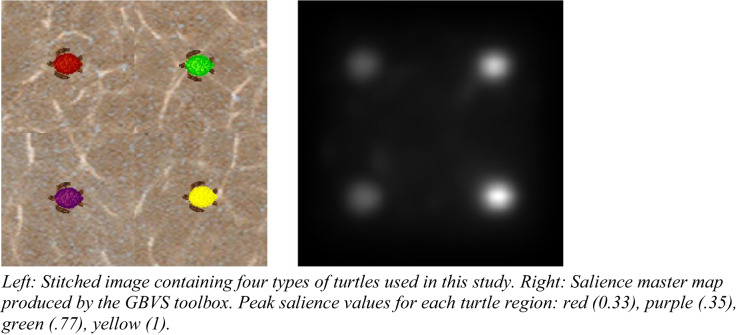
Normalized salience of turtle stimuli

**Fig. 11:**
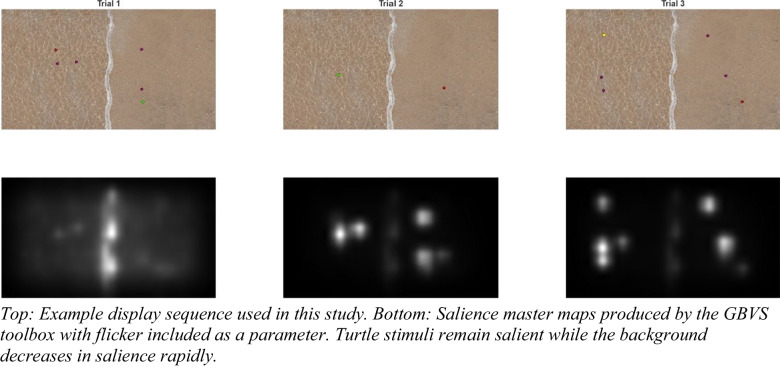
Salience of the middle wave and water reflection background

## Data Availability

All relevant data have been made accessible via the Open Science Framework (link: https://osf.io/rjsbu/).

## Appendix A

### Stimuli salience metrics (Graph-based Visual Saliency (GBVS))

The goal was to validate the assumption that free-viewing behavior would be driven by salience with an independent measure provided by the Graph-based Visual Saliency (GBVS) toolbox (Harel et al., 2007), a widely used saliency toolbox in the visual attention literature (e.g., Henderson et al., 2018; Kiat et al., 2022). The results validated the prediction that the high-saliency turtles would be looked at more frequently than other turtles under free viewing.

First, we ran an unnormalized comparison of eight 128 × 128 patches which contained just one turtle each (~35 × 30px) and its surroundings to get a measure of its peak salience. We used the GBVS MATLAB package to compute raw feature maps and activation maps. The feature maps used were the DKL Color, Intensity, and Orientation channels. This analysis was run using default parameters except we omitted the final mat2gray rescaling function. Table A1 shows the peak salience value within the master map output for each turtle patch. The yellow and green turtles were indeed more salient than the red and purple turtles according to the GBVS toolbox Table 5.

**Table 5:** Unnormalized peak salience of turtle stimuli

Background	Turtle color			
	Yellow	Green	Red	Purple
Sand	.0185	.0154	.0077	.0083
Water	.0123	.0106	.0054	.0055

Next, we stitched the four turtle patches together into one image and ran a normalized measurement using the default GBVS function. Figure 10 shows the yellow and green turtle as more salient than the red and purple turtles. Both GBVS analyses consistently ranked the yellow turtle as being more salient than the green one. This would predict more eye movements should be directed at the yellow turtle compared to the green turtle under free-viewing conditions and that is what our eye-movement data showed (Fig. 3). In fact, the results under free viewing demonstrate that the more an item is salient in the GBVS metric, the more it attracts eye movements.

Finally, we ran an analysis to explore the impact of the waveline in the middle of the display, on saliency of the entire image. The middle waveline was most salient when we included the full display as input. However, after including flicker as a parameter within GBVS, what we found was that the middle wave quickly loses salience over just a few trials. Figure 11 shows that a sequence of just three trials leads to reduced salience of the background. This is a well-documented effect in the attentional capture literature. The “learned distractor suppression effect” literature has shown that a salient distractor when repeated over consecutive trials becomes suppressed and no longer produces an attentional capture effect (e.g. Gaspar & McDonald, 2014; Gaspelin & Luck, 2018; Wang & Theeuwes, 2018). Thus, it is reasonable to assume that, having repeated the wave exactly at the same location on every trial, we observed the same effect in our experiment. Whereas the wave might have caught the attention of the participants at the very start of the experiment, it likely became suppressed early on during the experiment. This interpretation is supported by our data showing that overall, first fixations at the wave were uncommon (< 5% of search trials). Similarly, the water reflection pattern was negligible in terms of salience compared to the turtle items. The background patterns may have been similarly suppressed (see Note on Table 2) Tables 6 and 7.

## Appendix B

**Table 6:** ANOVA table for Experiment 1

Effect	*Ndf*	*Ddf*	*F*	${{\eta }_{p}}^{2}$	*p*_*adj*_
Item type	2.49	94.80	87.74	.698	*** <.001
Item type * salient item color	3.61	137.04	38.91	.506	*** <.001
Item type * purple item set size	7.44	282.69	45.84	.547	*** <.001
Item type * task instruction	2.49	94.8	24.50	.392	*** <.001
Item type * salient item color * purple item set size	8.41	319.73	3.18	.077	*** <.001
Item type * purple item set size * task instruction	7.44	282.69	5.60	.128	*** <.001
Item type * salient item color * task instruction	3.61	137.04	.49	.013	.73
Item type * salient item color * purple item set size * task instruction	8.41	319.73	1.75	.044	.08
Salient item color	1	38	.05	.001	.83
Purple item set size	3	114	2.70	.066	* <.05
Task instruction	1	38	2.84	.070	.10
Salient item color * purple item set size	3	114	3.90	.093	* <.05
Purple item set size * task instruction	3	114	1.17	.030	.32
Salient item color * task instruction	1	38	.95	.024	.34
Salient item color * purple item set size * task instruction	3	114	1.12	.029	.34

Appendix B. Main effects and interactions that do not involve the item type factor are not meaningful, because their corresponding comparisons will be between the aggregated first fixation counts that have not categorized the fixations by item type. $\eta_p^2$ = partial eta-squared. **p* <.05. ****p* <.001

## Appendix C

**Table 7:** ANOVA table for Experiment 2

Effect	*Ndf*	*Ddf*	*F*	${{\eta }_{p}}^{2}$	*p*_*adj*_
Item type	3.08	117.16	102.07	.729	*** <.001
Item type * salient item color	3.34	127.11	22.14	.368	*** <.001
Item type * less-salient item color	2.44	92.57	61.24	.617	*** <.001
Item type * task instruction	3.08	117.16	22.57	.373	*** <.001
Item type * salient item color * less-salient item color	3.34	123.03	.96	.025	.42
Item type * less-salient item color * task instruction	2.44	92.57	19.12	.335	*** <.001
Item type * salient item color * task instruction	3.34	127.11	.73	.019	.55
Item type * salient item color * less-salient item color * task instruction	3.24	123.03	.63	.016	.61
Salient item color	1	38	.18	.005	.68
Less-salient item color	1	38	4.08	.097	.51
Task instruction	1	38	1.21	.031	.28
Salient item color * less-salient item color	1	38	4.19	.099	* <.05
Less-salient item color * task instruction	1	38	2.67	.066	.11
Salient item color * task instruction	1	38	.29	.008	.59
Salient item color * less-salient item color * task instruction	1	38	.24	.006	.62

Appendix C. Main effects and interactions that do not involve the item type factor are not meaningful, because their corresponding comparisons will be between the aggregated first fixation counts that have not categorized the fixations by item type. $\eta_p^2$ = partial eta-squared. **p*_adj_ <.05. ****p*_adj_ <.001

## Code availability

Analysis code in R and experiment code in Python have been made accessible via the Open Science Framework (link: https://osf.io/rjsbu/).
